# Rapid and accurate detection of peanut pod appearance quality based on lightweight and improved YOLOv5_SSE model

**DOI:** 10.3389/fpls.2025.1494688

**Published:** 2025-02-20

**Authors:** Zhixia Liu, Xilin Zhong, Chunyu Wang, Guozhen Wu, Fengyu He, Jing Wang, Dexu Yang

**Affiliations:** College of Engineering, Shenyang Agricultural University, Shenyang, Liaoning, China

**Keywords:** appearance quality, YOLOv5, lightweighting, ShuffleNetv2, peanut pod

## Abstract

**Introduction:**

With the escalating demands for agricultural product quality in modern agriculture, peanuts, as a crucial economic crop, have their pod appearance quality directly influencing market value and consumer acceptance. Traditionally, the visual inspection of peanut pod appearance quality relies heavily on manual labor, which is not only labor-intensive and inefficient but also susceptible to subjective judgments from inspectors, thereby compromising the consistency and accuracy of inspection outcomes. Consequently, the development of a rapid, accurate, and automated inspection system holds significant importance for enhancing production efficiency and quality control in the peanut industry.

**Methods:**

This study introduces the optimization and iteration of the YOLOv5s model, aiming to swiftly and precisely identify high-quality peanuts, peanuts with mechanical damage, moldy peanuts, and germinated peanuts. The CSPDarkNet53 network of the YOLOv5s model was substituted with the ShuffleNetv2 backbone network to reduce the model’s weight. Various attention mechanisms were explored for integration and substitution with the backbone network to enhance model performance. Furthermore, the substitution of various loss functions was investigated, with the Focal-EIoU loss function employed as the regression loss term for predicting bounding boxes, thereby improving inference accuracy.

**Results:**

Compared to the YOLOv5s network model, SSE-YOLOv5s boasts a mere 6.7% of the original model’s parameters, 7.8% of the computation, and an FPS rate 115. 1% higher. Its weight size is a mere 7.6% of the original model’s, while the detection accuracy and mean average precision (mAP) reach 98.3% and 99.3%, respectively, representing improvements of 1.6 and 0.7 percentage points over the original YOLOv5s model.

**Discussion:**

The results underscore the superiority of the SSE-YOLOv5s model, which achieves a maximum mAP of 99.3% with a minimal model size of 1. 1MB and a peak FPS of 192.3. This optimized network model excels in rapidly, efficiently, and accurately detecting the appearance quality of mixed multi-target peanut pods, making it suitable for deployment on embedded devices. This study provides an essential reference for multi-target appearance quality inspection of peanut pods.

## 1 Introduction

Peanut, an essential oil crop, finds widespread applications in daily life and industries (Sun et al., 2022). However, during harvesting, transportation, and storage, peanuts are highly susceptible to damage, mold, and sprouting, thereby compromising their quality and value (Zhao et al., 2023). Early peanut sorting largely relied on manual labor, which is time-consuming, labor-intensive, and inefficient. Nowadays, advancements in computer vision and machine learning technologies have presented novel solutions for real-time and automated inspection of agricultural product quality (El-Mesery et al., 2019). Traditional machine learning approaches have garnered valuable experiences in quality inspection and grading of agricultural products, including image segmentation (e.g., K-means clustering (Jia et al., 2023) and thresholding methods (He et al., 2023)), feature detection (e.g., SURF (Saraswat et al., 2024), KAZE (Qiu et al., 2021), and MSER (Li et al., 2020)), and pattern recognition (e.g., KNN (Gao et al., 2022), SVM (Cao et al., 2023), and BP neural networks (He et al., 2023)). Nevertheless, due to the complexity of image preprocessing and feature extraction, these methods often yield suboptimal detection performance.

Deep learning can automatically learn hierarchical features from different appearance representation regions, eliminating the need for manual feature extraction and classifier design, and exhibiting remarkable generalization and robustness. The utilization of Convolutional Neural Networks (CNNs) for detecting crop appearance quality has emerged as a new hotspot in smart agriculture research. Yadav et al. (2022) proposed an improved VGG-16 model and developed SoftMax for classification, achieving an average accuracy of 99.84% in detecting citrus peel diseases. Dogra et al. (2023) developed a CNN-VGG19 model for identifying rice brown spot disease, leveraging transfer learning to achieve a maximum accuracy of 93.0%. Si et al. (2023) introduced a method based on weight contrast transfer and MobileNetV3 for detecting surface defects on apples, achieving a recognition accuracy of 94.47%. However, these methods are primarily suited for simple classification tasks. For complex detection tasks, the mainstream approach currently in use is YOLO. Su et al. (2022) proposed an SE-YOLOv3-MobileNetV1 network model for detecting four tomato maturity stages, achieving an average accuracy of 97.5%. Wang et al. (2022) presented an improved YOLOv4-Tiny algorithm for accurate and rapid recognition of sugarcane stem nodes, with an average precision of 99. 11%. Wang et al. (2023) designed a novel method based on modified YOLOv5 for grading kiwifruit defects, incorporating SPD-Conv and DW-Conv modules and using EIOU as the loss function. Results showed that the improved model reduced training loss by 0.013, achieved an overall detection accuracy of 97.7%, and required 8.0 ms for detection. Zhan et al. (2023) proposed the YOLOv5s_AMM model, replacing the original C3 network in YOLOv5s with the M3-Net from MobileNetV3, achieving rapid and accurate detection of walnut appearance quality with a maximum average detection accuracy of 80.78%, a reduced model size of 20.9 MB, and a detection speed of 40.42 frames per second. Zhang et al. (2023) introduced a YOLOv7-based neural network model for inflorescence recognition, incorporating an Efficient Multi-scale Attention Mechanism (EMA) and a parallel processing strategy to achieve cross-channel feature interaction, maximizing the retention of pixel-level features and positionalinformation on feature maps. Under varying time periods, distances, and weather conditions, the average detection accuracy and recall rate for inflorescence detection reached 91.4% and 89.8%, respectively. Uddin et al. (2024) employed an improved YOLOv8 to design the Cauli-Det model for automatic classification and localization of cauliflower diseases, demonstrating that the modified YOLOv8 model achieved an average precision of 91. 1% on the test dataset.

The above represents recent applications of machine learning and deep learning in agricultural product quality inspection. Notably, deep learning methods have also made significant contributions to peanut quality inspection. Wang et al. (2023) proposed a multi-spectral system combined with an improved Faster RCNN method for detecting peanut defects (mildew, mechanical damage, and germinated seeds). The results indicated that all evaluation metrics were improved compared to the original network, with the average detection accuracy (mAP) reaching 99.97% when using the VGG16 backbone network. Yang et al. (2023) introduced a peanut pod quality detection algorithm (PQDA) based on a modified ResNet convolutional neural network. They determined ResNet18 as the optimal backbone feature extraction network for model training and incorporated the KRSNet module, CSPNet module, and Convolutional Block Attention Module (CBAM) into the algorithm. The results showed that the improved PQDA model achieved an accuracy of 98. 1% with a parameter size of 32.63 MB. Wang et al. (2022) proposed the CG-SqueezeNet model for peanut pod quality grading. By introducing a coordinated attention module into the SqueezeNet model and adopting a gradient-focused optimization algorithm, the CG-SqueezeNet model achieved a detection accuracy of 97.83% with a parameter memory of 2.52 MB. Yang and Qin (2022) conducted preprocessing on RGB images of peanut kernels, extracted six color feature values, constructed a feature vector combining color and texture, and then classified peanut kernels using BP neural networks and SVM classifiers. The results revealed that the BP neural network achieved an overall recognition accuracy of 96.67%, while the SVM classifier achieved 97.22%. Zhang et al. (2020) proposed a peanut quality inspection method based on machine vision and an adaptive convolutional neural network to identify common defects such as mold, breakage, and shriveling. To enhance the model’s generalization ability, a transfer learning algorithm was introduced. The results showed that this method achieved an average recognition rate of 99.7% for common peanut defects. Jiang et al. (2024) presented a terahertz wave imaging system using a convolutional neural network (CNN) machine learning approach for identifying standard, moldy, defective, dried, and germinated peanut seeds. The average detection time was 2.2 seconds, with a recognition accuracy of 98.7%. Lin et al. (2022) combined an improved YOLOv5s, DeepSort, and OpenCV programs to propose a real-time video counting model for peanuts. The results demonstrated an accuracy of 98.08%.

As aforementioned, numerous scholars have proposed highly innovative ideas and methods in the field of agricultural product quality inspection, which have been successfully applied in agricultural production, yielding remarkable results. However, there remains room for further improvement in peanut quality inspection, particularly in addressing challenges such as the small size of moldy peanut targets and overlapping during detection. Many existing methods achieve high detection accuracy but come with significant computational costs, or vice versa. This study aims to develop a model that combines high detection precision with low parameter counts. It endeavors to extract peanut appearance quality feature information from various angles and structures, leveraging different feature representations. By fusing feature structures that embody distinct semantics, the study seeks to enhance the detection quality of peanut pod appearance. The refined model has undergone continuous comparative experiments to achieve the desired performance.

## 2 Materials and methods

### 2.1 Image acquisition

**Figure 1:**
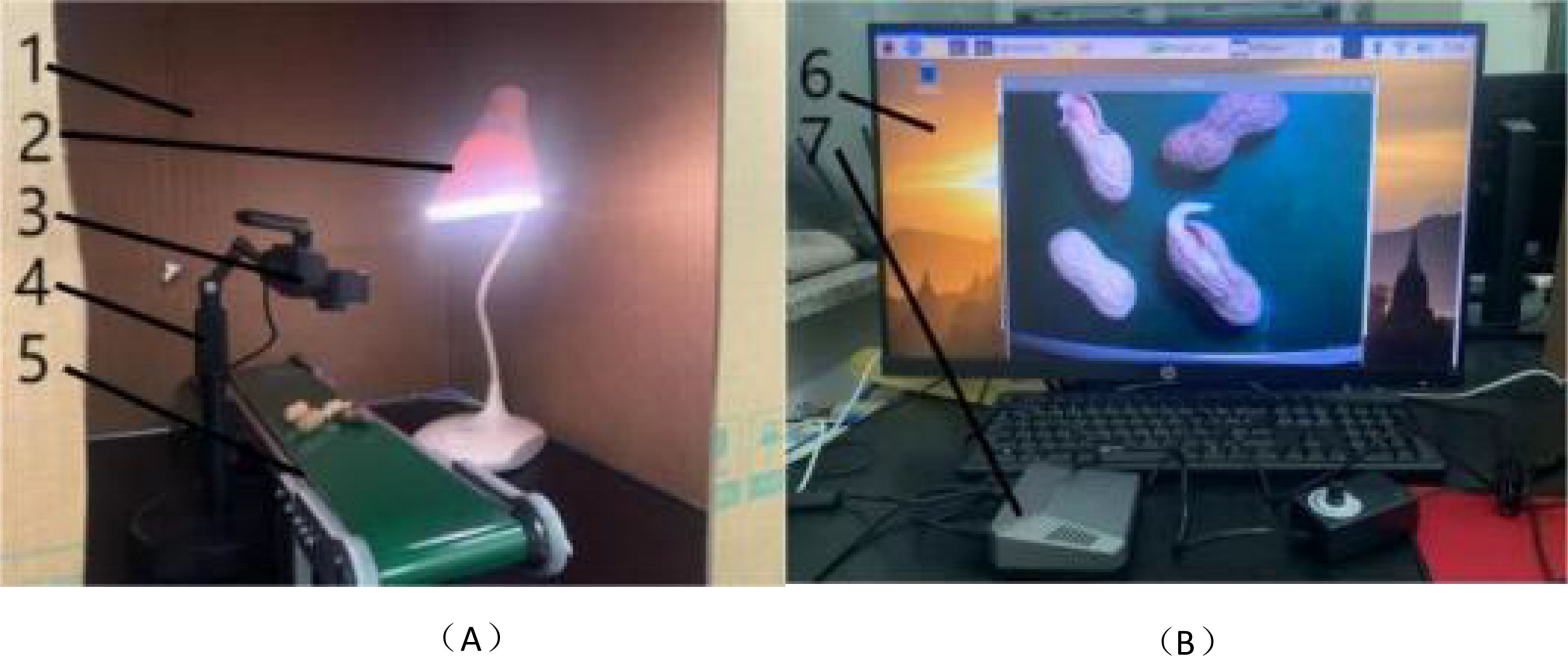
Illustration of the **(A)** acquisition equipment **(B)** image display. 1-Dark Box Enclosure, 2- Lighting Sources, 3- Camera, 4- Camera Mount, 5- Conveyor Belt, 6- Operation Platform, 7- Raspberry Pi.

The experiments were conducted in the Engineering Foundation Laboratory at Shenyang Agricultural University, Shenyang, Liaoning Province, China. Three types of peanut samples (Tieyinhua No.1, Qinghua 308, and Nihon Dou) were collected from the Tieling Academy of Agricultural Sciences, Liaoning Province. Image acquisition was performed between 9:00 AM and 6:00 PM from March 10th to March 15th, 2024. As depicted in **Figure 1**, the image acquisition setup consisted of a dark box enclosure, lighting sources, a camera, a camera mount, a conveyor belt, an operation platform, and a Raspberry Pi. The camera (Webcam) featured 2 megapixels with a maximum resolution of 1280×720, capturing images in JPEG format. The conveyor belt had overall dimensions of 500×100×73 mm, operated with an adapter voltage of 110-230V, and offered a speed range from 1.8 cm/s (slowest) to 7.6 cm/s (fastest). The Raspberry Pi 4B employed a Broadcom BCM2711 SOC, powered by a 64-bit 1.5 GHz quad-core CPU. The camera was securely mounted at a height of 150 mm above the desktop, while the light sources were positioned 220 mm above the desktop. All images were captured under uniform conditions, including consistent camera height, even light source brightness, and a standardized background. A total of 1600 images were acquired, with 320 images captured daily.

### 2.2 Image data augmentation

**Figure 2:**
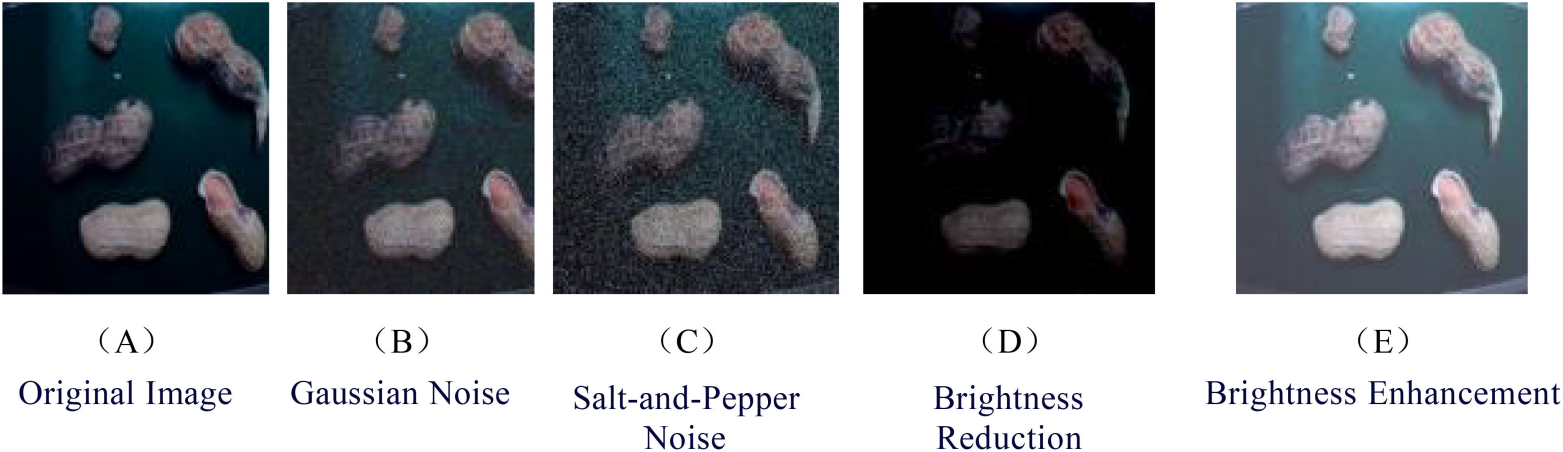
Data augmentation images. **(A)** Original Image **(B)** Gaussian Noise **(C)** Salt-and-Pepper Noise **(D)** Brightness Reduction **(E)** Brightness Enhancement.

Using Python software, the 1600 original images underwent image data augmentation, resulting in an expansion to 8000 images through techniques such as Gaussian noise, salt-and-pepper noise, brightness reduction, and brightness enhancement. These images were then sliced and segmented into uniform-sized images of 640 pixels × 640 pixels, as depicted in **Figure 2**. Annotation was performed using the LabelImg tool, generating VOC-formatted.xml files, which were subsequently converted into YOLO-formatted.txt files. Finally, the annotated images were randomly divided into a training set of 5600 images, a validation set of 1600 images, and a test set of 800 images, according to a 7:2:1 ratio.

### 2.3 YOLOv5 network model and optimized architecture

#### 2.3.1 YOLOv5 model

YOLOv5, compared to previous YOLO series object detection models, places greater emphasis on small object detection while maintaining high accuracy and speed. YOLOv5 offers a total of five versions, ranging from smallest to largest in model size: YOLOv5n, YOLOv5s, YOLOv5m, YOLOv5l, and YOLOv5x. These models differ in width and depth, enabling YOLOv5 to adapt to various datasets and facilitating user selection.

**Table 1** provides a comparison of detection accuracy, model size, and detection performance among four different YOLOv5 models. In terms of detection accuracy, YOLOv5s exhibits a slightly lower precision (P) compared to YOLOv5m (by 0.06%), YOLOv5l (by 1.5%), and YOLOv5x (by 1.8%). However, when considering model size, YOLOv5s stands out due to its compact size of 16.3 MB, significantly smaller than YOLOv5m, offering a reduction of 34.3 MB. This size advantage makes YOLOv5s an economically efficient choice, particularly crucial for embedded devices with storage constraints. In terms of detection speed, YOLOv5s outperforms other models, processing 93.17 more frames per second (FPS) than YOLOv5m, 105.35 more FPS than YOLOv5l, and 112.96 more FPS than YOLOv5x. YOLOv5s’ superior inference speed positions it as an excellent choice for real-time detection scenarios and applications requiring quick responses. Given YOLOv5s’ emphasis on low-latency and cost-effective deployment for lightweight multi-object detection within Peanut pods, it presents a compelling proposition with a detection accuracy of 97.7%, a model size of 14.4 MB, and fast detection speed.

**Table 1:** Comparison of performance among different YOLOv5 models.

Model	P(%)	R(%)	mAP0.5(%)	FPS(.s-1)	Model Size(MB)	FLOPs(G)	Parameters
YOLOv5s	96.7	95.4	97.7	166.70	14.4	16.3	7074330
YOLOv5m	96.7	96.2	98.3	73.53	40.5	50.6	21072570
YOLOv5l	98.2	98.4	99.2	61.35	89.4	114.6	46652890
YOLOv5x	98.3	98.5	99.5	53.74	167	217.9	87271290

**Figure 3:**
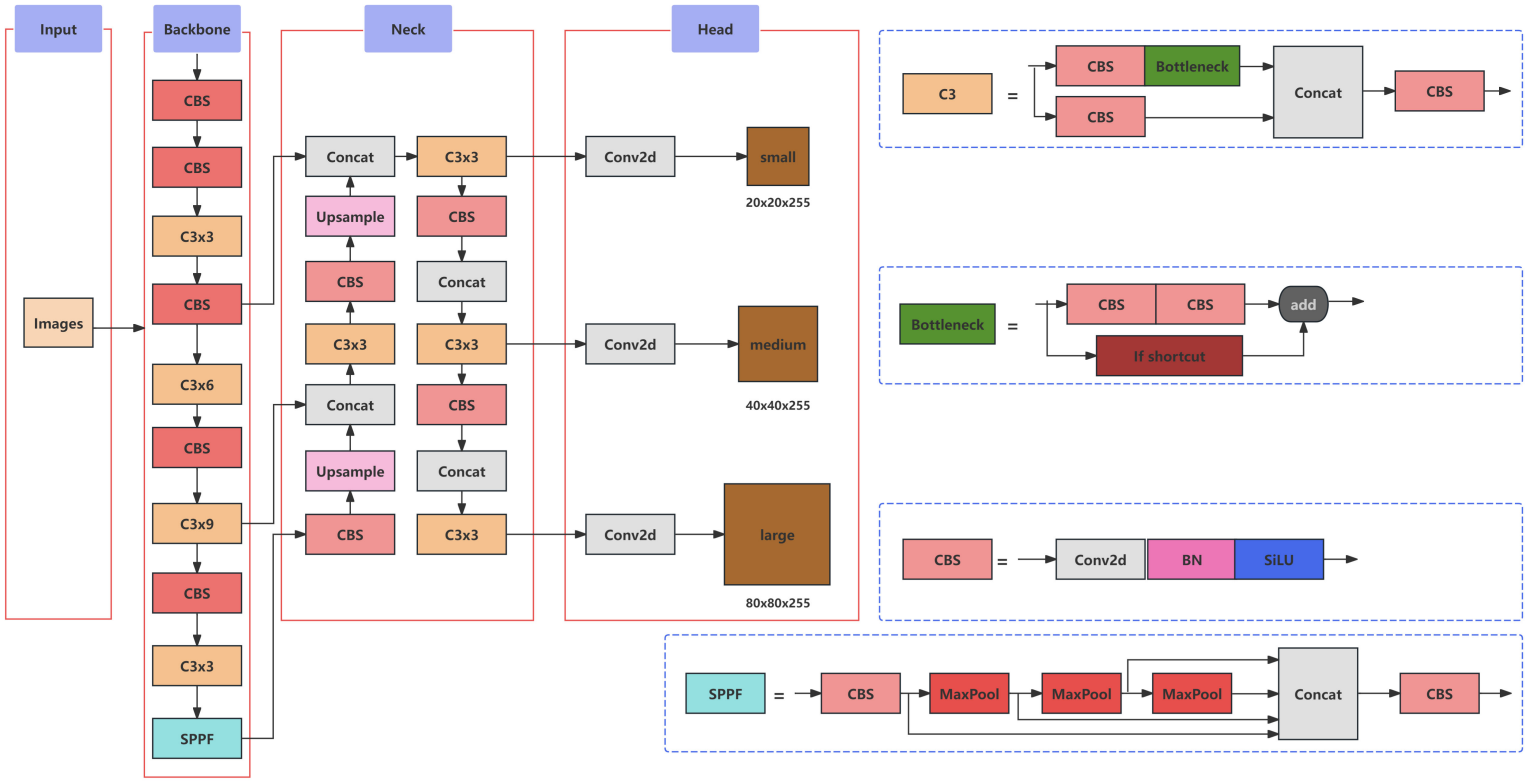
The architecture of the YOLOv5 model.

YOLOv5 is an object detection algorithm, whose model architecture primarily comprises an input stage, a Backbone network, a Neck network, an output stage, and an activation function. The input stage, which primarily facilitates image preprocessing, incorporates Mosaic image enhancement, adaptive anchor box calculation, and adaptive image resizing. The Backbone layer, composed mainly of the CSPResNet53 (Bochkovskiy et al., 2020) structure, is responsible for feature extraction. YOLOv5 employs two distinct Neck network structures: SPPF (Spatial Pyramid Pooling Fast) (He et al., 2014) and PAN (Path Aggregation Network) (Jiang et al., 2019). The SPPF structure enhances the model’s perception capability and scale invariance, while the PAN structure strengthens the fusion of multi-scale features. The output stage of YOLOv5 primarily consists of prediction boxes, where each box comprises confidence, class probabilities, and bounding box coordinates. YOLOv5 utilizes anchor boxes in the output layer to predict the position and size of object bounding boxes, while the softmax function is employed to calculate class probabilities for each prediction corresponding to an anchor box. Mish, an activation function that serves as a substitute for ReLU, is used in YOLOv5 to enhance model performance. The architecture of the YOLOv5 model is illustrated in **Figure 3**.

#### 2.3.2 ShuffleNetv2 lightweight backbone network

To achieve a lightweight network architecture for enhancing YOLOv5s in detecting the external quality of peanut pods, ShuffleNet v2 is adopted as the backbone network. ShuffleNet v2 (Ma et al., 2018), proposed by Megvii in July 2018, strikes a balance between speed and accuracy. Distinct from ShuffleNet v1 (Zhang et al., 2018), ShuffleNet v2 adheres to the G1-G4 guidelines as closely as possible while designing an efficient network that excels in both speed and accuracy.

**Figure 4:**
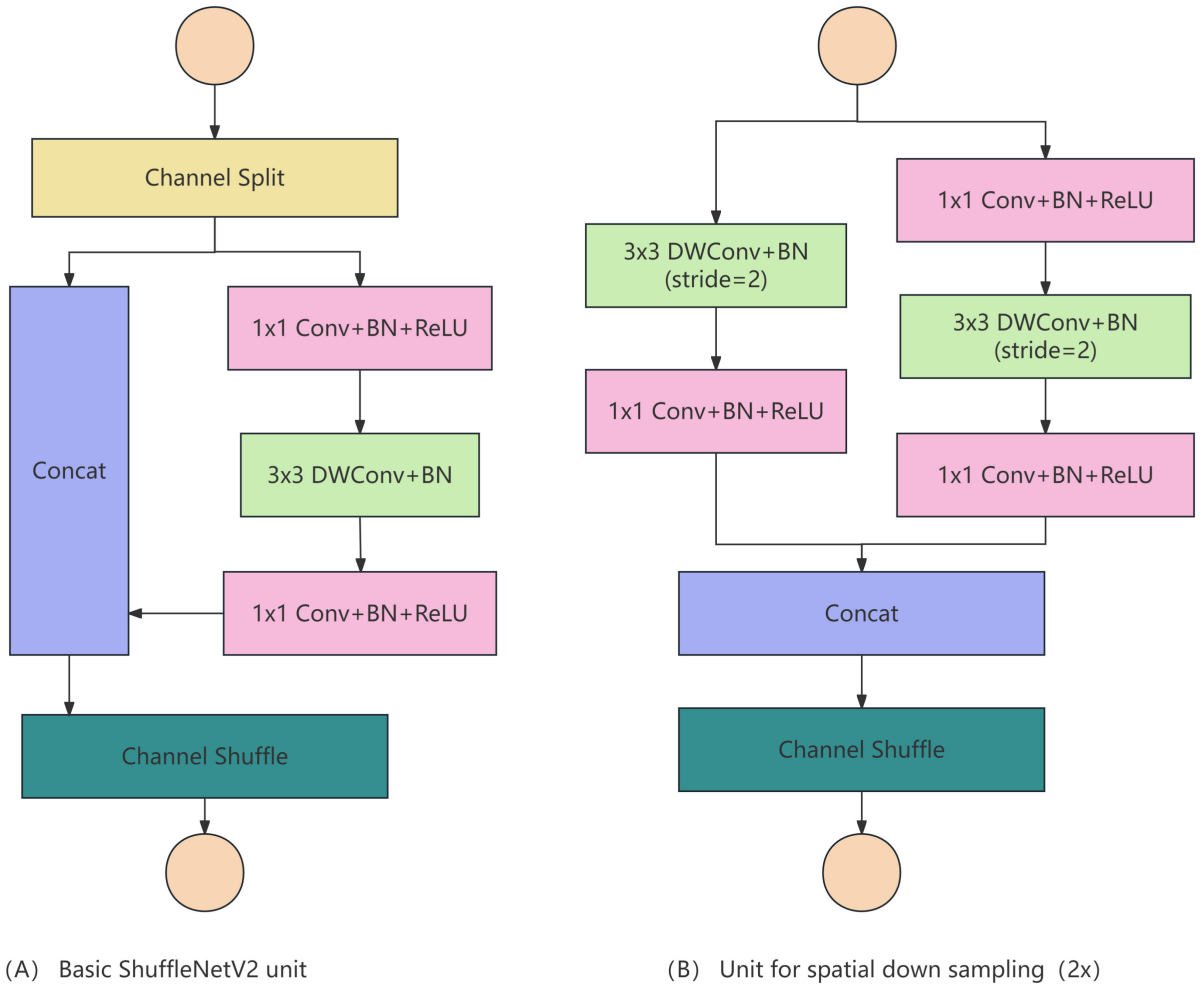
**(A)** Basic Block of ShuffleNet v2 **(B)** Spatial Downsampling Module.

The fundamental block of ShuffleNet v2, as depicted in **Figure 4A**, employs channel split (Weng et al., 2019) to divide the input feature maps into two branches with equal channel numbers along the channel dimension. The left branch serves as an identity mapping to reduce network fragmentation, while the right branch comprises three convolutions with identical channel numbers, ensuring that the input and output channels remain the same. A Concat operation is then performed on these two branches, resulting in output feature maps with the same channel count as the original input. Channel shuffle is utilized to facilitate information exchange between the two branches. The spatial downsampling module (He et al., 2016), illustrated in **Figure 4B**, eliminates channel split and utilizes the original feature maps for both branches. By modifying the stride of the convolutional units to 2, the concatenated feature maps are halved in size while doubling the channel count.

#### 2.3.3 SE attention mechanism

**Figure 5:**
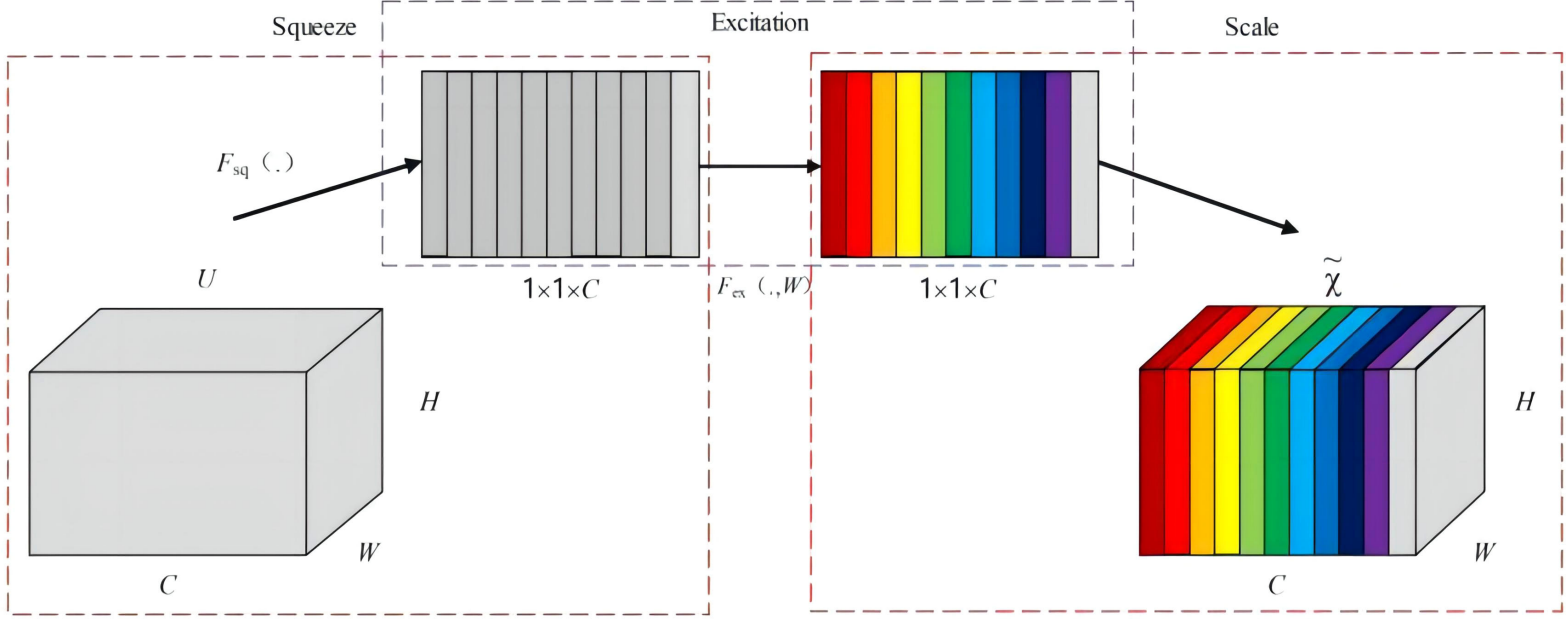
Basic structure of the SE module.


SE Net (Hu et al., 2020) (Squeeze-and-Excitation Networks) represents a channel attention mechanism that imposes weights on different channel features through a learning-based approach at the channel level. A higher weight indicates greater relevance, assisting the model in capturing the significance of each channel, emphasizing useful information while ignoring irrelevant details, thereby enhancing the accuracy of detection outcomes. The SE network initially employs a global information embedding (Squeeze) operation, as illustrated in **Figure 5**, to perform global pooling on the original 3D feature maps of size C×W×H. This compression function Fsq sequentially traverses the height and width of each feature map. Consequently, the compression calculation can be formulated as compressing each 2D feature channel (W×H) into a single real number along the spatial dimension, with the output dimension matching the number of input feature channels, yielding a feature map with a global receptive field of 1×1×C, as expressed in Equation 1.

(1) $${\text{F}}_{\text{sq}}({\text{u}}_{\text{c}})={\text{z}}_{\text{c}}=\frac{1}{\text{H}\times \text{W}}\sum_{\text{i}=1}^{\text{H}}\sum_{\text{j}=1}^{\text{W}}{\text{u}}_{\text{c}}(\text{i},\text{j})$$

In the equation, Fsq represents the global average pooling operation applied across channels; uc denotes the c-th 2D matrix of the 3D feature map u; Zc is the c-th element of Z; and W×H refers to the dimensions of the 2D feature channel.

The second step is the adaptive recalibration (Excitation), where the module consists of two sequentially connected fully connected neural networks. It employs a sigmoid function to generate weights for each feature channel, signifying the correlations among various feature channels, as calculated in Equation 2.


(2) $$F_{ex}(z,W)=sigmoid[W_{2}\times Re\;LU(W_{1}z)]$$

In the equation, Fex represents the adaptive recalibration operation; W1 and W2 denote the linear layers; z is the feature map after the Squeeze operation.

The final Scale operation assigns weights to each of the c channels, accomplishing adaptive recalibration of the original features in the channel dimension. This procedure enhances salient features while attenuating less critical ones, resulting in varying levels of importance across individual channels. This, in turn, enhances the directivity of the extracted features, yielding the final feature map as presented in Equation 3.


(3) $$F_{scale}(u_{c},s_{c})={\tilde{x}}_{c}=s_{c}u_{c}$$

In the equation, Fscale represents the channel-wise weighting of the weights; $\tilde{x}$ leads to the final output; sc denotes the channel-specific weight.

The application of the SE Net attention mechanism can, to a certain extent, compensate for the loss in detection accuracy incurred by lightweight feature extraction networks. The attention mechanism enhances the model’s sensitivity to targets, enabling it to disregard background features and improve recognition accuracy.

#### 2.3.4 Loss function

**Figure 6:**
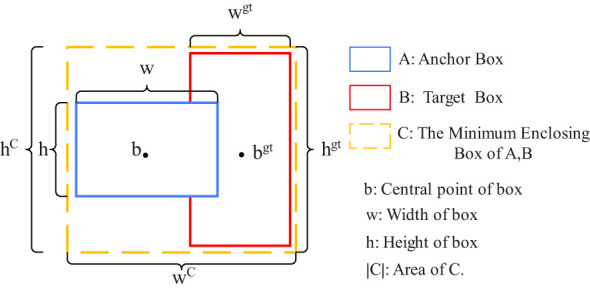
Schematic diagram of the loss function.


The loss function measures the discrepancy between the predicted results and the true labels, with a smaller value indicating a closer prediction to the actual label. Given the high similarity between peanut pods and soil clumps, particularly the inaccurate localization of small targets, the Focal-EIoU (Lin et al., 2020) boundary box regression loss function is introduced to enhance the model’s localization capability. A schematic diagram of the loss function is presented in **Figure 6**. The EIoU (Efficient Intersection over Union) (Lin et al., 2020) algorithm ingeniously balances the contribution of high- and low-quality samples to the loss function by incorporating positional information and redesigning the calculation of penalty terms. This innovation not only effectively reduces the likelihood of free transformation of predicted bounding boxes during training but also guides the detection boxes to approach the true target boxes with more precision and rationality, thereby significantly enhancing the regression accuracy in object detection tasks. The EIoU loss function, denoted as LEIOU, is defined as follows:

(4) $$\begin{array}{l} L_{EIOU}=\;L_{IOU}+L_{dis}+L_{asp}\\ \;=1-IOU+\frac{\rho^{2}(b,b^{gt})}{{\left(w^{c}\right)}^{2}+{\left(h^{c}\right)}^{2}}+\frac{\rho^{2}(w,w^{gt})}{{\left(w^{c}\right)}^{2}}+\frac{\rho^{2}(h,h^{gt})}{{\left(h^{c}\right)}^{2}} \end{array}$$

In the equation, LEIOU represents the EIoU loss function; LIOU denotes the overlap loss between the predicted bounding box and the ground truth box; Ldis represents the center distance loss between the predicted bounding box and the ground truth box; Lasp represents the width and height loss between the predicted bounding box and the ground truth box; hC and wC are the width and height of the smallest enclosing rectangle that contains both the predicted and ground truth boxes; b represents the center coordinates of the predicted bounding box; bgt represents the center coordinates of the ground truth box; h and w are the width and height of the predicted bounding box, respectively; and wgt and hgt are the width and height of the ground truth box, respectively.

In the context of bounding box regression, high-quality anchors are significantly outnumbered by low-quality ones, posing a detrimental effect on the training process. Consequently, there is a pressing need to investigate strategies that can amplify the influence of high-quality anchors. To direct the EIoU loss function towards greater emphasis on high-quality samples, we propose the Focal-EIoU Loss, denoted as LFocal-EIoU, which is defined as follows:


(5) $$L_{Focal-EIOU}=IOU^{Y}L_{EIOU}$$

In the equation,γ=0.5.

#### 2.3.5 Improving the network

##### 2.3.5.1 Architecture of YOLOv5

**Figure 7:**
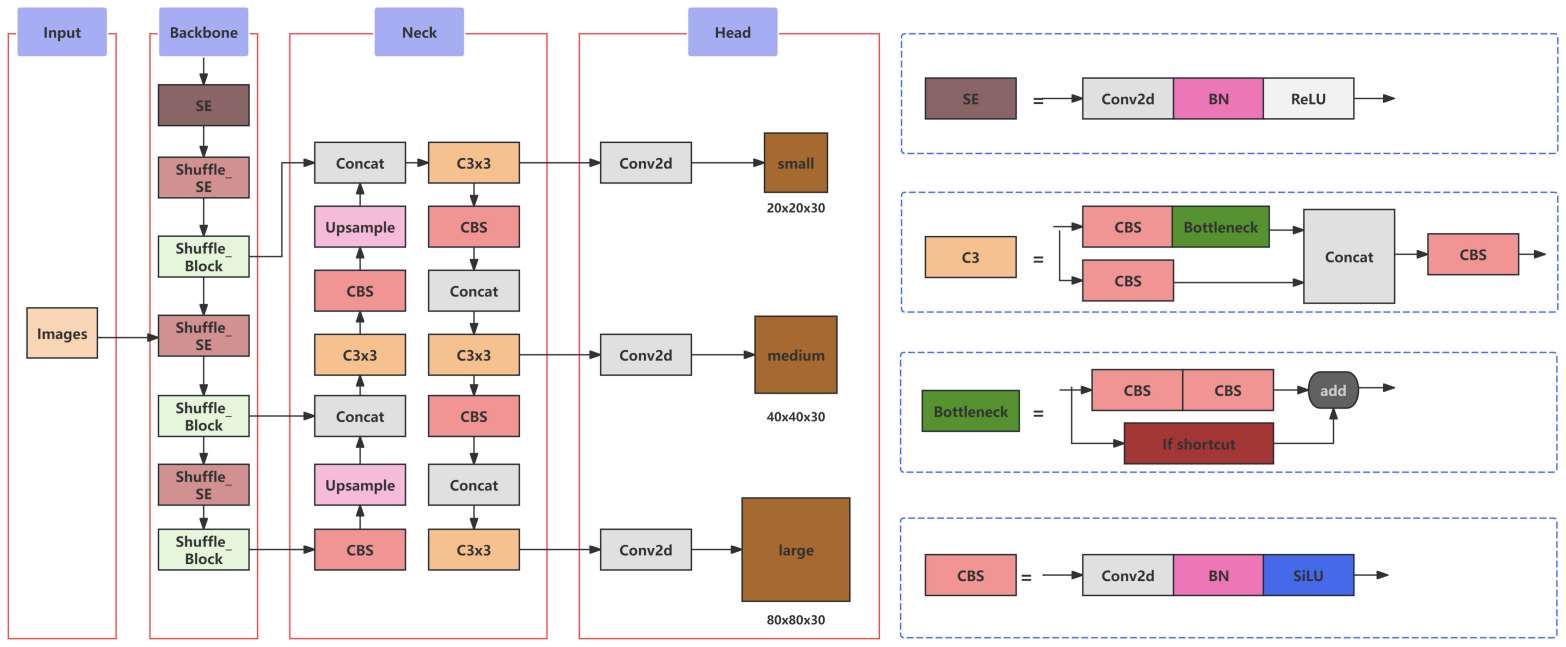
Improved network architecture of YOLOv5.

In this study, a YOLOv5-based architecture was established as the foundation for model improvement, aiming to address issues related to accuracy, model size, and detection speed, thereby developing a more suitable model for detecting peanut pod categories during the primary processing stage. The overall enhanced network structure is illustrated in **Figure 7**. Within this model refinement, the CSPDarkNet53 network of the original YOLOv5 was selected to be replaced by the ShuffleNetv2 backbone network. This alteration was undertaken to reduce model size while ensuring a lightweight and efficient design. Furthermore, the Convolutional Attention Module, SE Net (Squeeze-and-Excitation Networks), was introduced into the backbone layers to compensate for any loss in detection accuracy incurred by the lightweight feature extraction network. Lastly, the Focal-EIoU loss function was adopted as the regression loss metric for bounding box predictions, enhancing inference accuracy. This adjustment was tailored to improve the input-specific data distribution, ultimately strengthening feature detection across varying image scales.

## 3 Results

### 3.1 Model specifications

The computer specifications include an Intel Core i7-12700F Central Processing Unit operating at a frequency of 2.10 GHz. It is equipped with 32GB of RAM and an NVIDIA GeForce RTX 3060 Ti graphics card, featuring 8GB of dedicated video memory. The development environment runs on Windows 10 operating system, accompanied by the PyTorch deep learning framework, PyCharm IDE, Python 3.8 configuration, and a Raspberry Pi 4B device.

**Table 2** provides a detailed overview of the model training specifications. Commencing with an initial learning rate of 0.01, the learning rate is subsequently reduced using a cosine annealing schedule. The neural network parameters are optimized via Stochastic Gradient Descent (SGD) with a momentum parameter set to 0.937 and a weight decay factor of 0.0005.

**Table 2:** Training program parameter settings.

Parameter	Batch size	Epochs	Input size	Optimizer	Initial learning rate	Momentum	Weight decay
Value	16	100	640x640	SGD	0.01	0.937	0.0005

Each training batch contains 16 images, and the training process spans 100 epochs, with input images resized to 640 × 640 pixels.

### 3.2 Evaluation metrics

The selection of Precision (P), Recall (R), F1 Score, and Mean Average Precision (mAP) as evaluation metrics for assessing the accuracy of object detection is based on the following calculation formulas, respectively:


(6) $$P=\frac{\text{TP}}{\text{TP}+\text{FP}}$$

(7) $$R=\frac{\text{TP}}{\text{TP}+\text{FN}}$$

(8) $$F1=2\times \frac{PR}{P+R}$$

(9) $$\text{mAP}=\frac{1}{N}\sum_{i=1}^{N}\int_{0}^{1}p_{i}(R)dR$$

Wherein, TP (true positive), FP (false positive), and FN (false negative) represent the number of correctly predicted, falsely predicted, and missed peanut pod targets, respectively; N denotes the number of detection categories, which is 5 in this study; pi(R) represents the PR curve for each category plotted using precision and recall. The Intersection over Union (IoU) is used to determine the closeness between the predicted bounding box and the ground truth box. When the IoU between the predicted box and the ground truth box is greater than 0.5, the detection result is considered correct, and the resulting mAP value is denoted as mAP0.5. The complexity of the model is represented by the number of parameters and floating-point operations per second (FLOPs), while the detection speed of the model is evaluated using frames per second (FPS).

### 3.3 Experimental results of the SSE-YOLOv5 Model

The experimental results of the SSE-YOLOv5 model are presented in **Table 3**. It can be observed that the improved YOLOv5 model achieves mean average precision (mAP0.5) of 99.6%, 99.6%, 99.5%, 98.6%, and 99.2% for the five categories of Good, Loss, Germinant, Moldy, and Soil, respectively. Notably, the highest average detection accuracy is achieved for Good and Loss categories, with all five categories demonstrating over 95% detection accuracy. This indicates that the SSE-YOLOv5 model exhibits high detection accuracy for the visual quality assessment of peanut pods and can be effectively utilized for this purpose.

**Table 3:** Experimental results of the SSE-YOLOv5 model.

	Good	Loss	Germinant	Moldy	Soil
mAP0.5/%	99.6	99.6	99.5	98.6	99.2
R/%	99.9	98.6	98.8	95.4	99.8
P/%	97. 1	98.9	98.7	97.9	98.9

### 3.4 Results of ablation experiments

To specifically verify the impact of each improvement on the network model, a detailed ablation study was conducted on the feasibility and effectiveness of ShuffleNetv2 lightweight network, SE attention mechanism, and Focal-EIoU loss function. The results of the lightweight network ablation experiments are presented in **Table 4**.

**Table 4:** Ablation study results of network lightweighting.

ShuffleNet v2	SE attention	Focal-EIoU loss function	mAP0.5/%	Parameters/MB	FLOPs/G	FPS/(. s−1)	P/%	R/%	MODEL SIZE/MB
–	–	–	98.6	7.07	16.3	166.7	96.7	95.4	14.4
√	–	–	97.2	0.44	1.3	173.8	94. 1	92.2	1.0
–	√	–	99.2	7.27	16.6	166.9	98. 1	98. 1	14.4
–	–	√	99. 1	7.06	16.3	165.1	98.2	97.6	14.4
√	√	–	98.5	0.46	1. 1	196.1	97. 1	94.6	1. 1
√	–	√	98.3	0.44	1.3	181.8	95.4	94.9	1.0
–	√	√	99.6	7.07	16.6	175.4	99.0	99.0	14.4
√	√	√	99.3	0.47	1.3	192.3	98.3	98.5	1. 1

Replacing the backbone of the original YOLOv5s with the ShuffleNetv2 lightweight network resulted in a 1.4% decrease in mAP0.5 compared to the original YOLOv5s. Although the detection accuracy was lower, it demonstrated that ShuffleNetv2 possesses a certain degree of feature extraction capability. By introducing depthwise separable convolutions, a significant reduction in network parameters and computational complexity was achieved, realizing network lightweighting and enhancing detection speed. Additionally, the network utilizing ShuffleNetv2 exhibited a 7.1 frames per second (FPS) higher detection rate for peanut pods compared to the original YOLOv5s, with a model size of merely 1.0MB, indicating the advantages of ShuffleNetv2 in embedded device applications.

When SE attention mechanism was solely incorporated into the backbone network or only the loss function was changed to Focal-EIoU, there were respective increases of 0.6% and 0.5% in mAP0.5 compared to the original YOLOv5s. These results indicate that the introduction of attention mechanisms and the use of Focal-EIoU loss function facilitate the network in extracting critical features of peanut pods, thereby enhancing detection accuracy.

Combining the SE attention mechanism in the backbone network with the Focal-EIoU loss function led to a 1.0% increase in mAP0.5 compared to the original YOLOv5s.

Embedding the SE attention module within ShuffleNetv2 as the feature extraction network resulted in a 1.3% improvement in mAP0.5 and a 3.0% boost in detection accuracy compared to ShuffleNetv2 alone, with a 22.3 FPS increase. This underscores the effectiveness of integrating SE attention modules within ShuffleNetv2 in enhancing both detection speed and accuracy while reducing model complexity.

Substituting the backbone of the original YOLOv5s with ShuffleNetv2 and replacing the loss function with Focal-EIoU not only improved the model’s computational speed but also enhanced mAP0.5 by 1. 1% and FPS by 8 frames compared to ShuffleNetv2 alone.

Based on comprehensive ablation studies, and aiming to balance model lightweighting with recognition accuracy, this study proposes an improved lightweight peanut pod appearance quality detection model based on YOLOv5s, named SSE-YOLOv5s, which incorporates ShuffleNetv2 + SE attention + Focal-EIoU loss function. Compared to the YOLOv5s network model, SSE-YOLOv5s boasts a mere 6.7% of the original model’s parameters, 7.8% of the computational complexity, 115. 1% of the FPS, and 7.6% of the model size. Furthermore, it achieves precision and mean average precision (mAP) of 98.3% and 99.3%, respectively, representing a 1.6% and 0.7% improvement over the original YOLOv5s model. Thus, SSE-YOLOv5s successfully achieves lightweighting while enhancing the model’s recognition accuracy for peanut pod quality.

### 3.5 Influence of various backbone networks on detection performance

To analyze the effect of network models on enhancing the performance of different backbone networks, the CSPResNet53 backbone in YOLOv5s was replaced with ShuffleNet v2, MobileNetv3 (Howard et al., 2019), and GhostNet (Rajaraman et al., 2018) respectively. The comparison results are presented in **Table 5**.

**Table 5:** Impact of various backbone networks on detection performance.

Backbone	mAP0.5/%	P/%	R/%	F1 score/%	Parameters/MB	FLOPs/G	FPS/(.s−1)
CSPResNet53	98.6	96.7	95.4	96.0	7.07	16.3	166.7
ShuffleNet v2	97.2	94. 1	92.2	93.1	0.44	1.3	173.8
MobileNetv3	97.8	93.6	93.6	93.4	3.54	6.3	138.7
GhostNet	98.8	96.8	96.2	96.4	5.08	10.6	151.9

As observed in **Table 5**, while utilizing the ShuffleNet v2 backbone led to a certain degree of compromise in detection accuracy, it significantly reduced the number of parameters and computations compared to the other models. Notably, it achieved the highest detection speed of 173.8 frames per second (FPS). Consequently, employing the ShuffleNet v2 backbone network is more conducive to lightweight model applications.

### 3.6 Impact of various attention mechanisms on detection performance

To investigate the performance of network models with the incorporation of different attention mechanisms, the SE (Squeeze-and-Excitation) attention module in the modified YOLOv5s was replaced with CBAM (Convolutional Block Attention Module) (Woo et al., 2018), ECA (Efficient Channel Attention) (Wang et al., 2020), and CA (CoordAttention) (Hou et al., 2021). The comparative results are summarized in **Table 6**.

**Table 6:** Impact of various attention mechanisms on detection performance.

Attention mechanisms	mAP 0.5/%	P/%	R/%	F1 score/%	Parameters/MB	FLOPs/G	FPS/(.s−1)
CBAM	98.8	97. 1	96.2	96.6	7.27	16.6	155. 1
ECA	98.9	97.3	96.7	96.9	7.20	16.6	158.8
CA	98.6	97.5	95.2	99.0	7.26	16.6	103.4
SE	99.2	98. 1	98. 1	98. 1	7.27	16.6	166.9

As shown in **Table 6**, the model utilizing the SE attention mechanism achieved a mAP0.5 of 99.2%, outperforming CBAM, ECA, and CA by 0.4, 0.3, and 0.6 percentage points, respectively. Additionally, the model with SE attention demonstrated higher accuracy and recall rates compared to CBAM, ECA, and CA, by 1.0, 0.8, and 0.6 percentage points for accuracy, and 1.9, 1.4, and 2.9 percentage points for recall, respectively. Notably, the F1 score of the SE-attentive model was only slightly lower than that of CA, indicating that the channel-wise attention allocation by SE significantly enhanced the model’s performance. While the number of parameters slightly increased and the FLOPs (Floating-Point Operations) remained comparable when comparing SE with CBAM, ECA, and CA, the SE attention module exhibited an advantage in terms of FPS (Frames Per Second). Based on the above analysis, SE attention achieves the highest detection accuracy while maintaining network lightweightness, demonstrating the optimal performance for peanut pod detection.

### 3.7 Impact of various loss functions on detection performance

To investigate the performance of network models with different loss functions, the CIoU (Complete Intersection over Union) loss function in YOLOv5s was replaced with Alpha-IoU, EIoU (Efficient Intersection over Union), and Focal-EIoU (Focal-Efficient Intersection over Union) loss functions. The comparative results are presented in **Table 7**.

**Table 7:** Impact of various loss functions on detection performance.

Loss function	mAP0. 5/%	P/%	R/%	F1 score/%	Parameters/MB	FLOPs/G	FPS/(. s−1)
CIoU	98.6	96.7	95. 1	95.8	7.07	16.3	166.7
Alpha-IoU	98.7	96.8	96.2	96.4	7.06	16.3	159.8
EIoU	98.7	97.5	95.6	96.5	7.06	16.3	161.5
Focal-EIoU	99. 1	98.2	97.6	97.4	7.06	16.3	165. 1

As shown in **Table 7**, the model utilizing the Focal-EIoU loss function achieved a mAP0.5 of 99. 1%, outperforming CIoU, Alpha-IoU, and EIoU by 0.5, 0.4, and 0.4 percentage points, respectively. Furthermore, the number of parameters, FLOPs (Floating-Point Operations), and FPS (Frames Per Second) values of the model with Focal-EIoU loss were comparable to those of CIoU,

Alpha-IoU, and EIoU. This demonstrates that the adoption of the Focal-EIoU loss function significantly enhances the average detection accuracy. This improvement can be attributed to the inclusion of vector angle loss between the predicted and ground truth bounding boxes in Focal-EIoU, which reduces the loss incurred during the “wandering” process of prediction boxes and ultimately improves inference accuracy.

### 3.8 Comparison of results from different detection models

To further analyze and validate the effectiveness of the proposed method in this study, it was compared with representative two-stage object detection algorithms such as Faster R-CNN (Ren et al., 2015), and one-stage object detection algorithms including SSD (Liu et al., 2016), YOLOv7 (Ultralytics, 2023), and YOLOv8. As shown in **Table 8**, although Faster R-CNN achieves a high accuracy in detecting the appearance quality of peanut pods, it has the largest number of parameters, making it unsuitable for lightweight applications. While SSD reduces the number of parameters compared to Faster R-CNN, its detection accuracy is not as high as the proposed method. YOLOv7 and YOLOv8 not only have relatively larger numbers of parameters and floating-point operations (FLOPs) compared to the proposed method, hindering lightweight design, but also generally perform inferiorly in terms of mAP0.5 and accuracy. The algorithm presented in this study, which is an improvement upon YOLOv5s, achieves a 0.7% increase in mAP0.5 over the baseline model YOLOv5s, with a reduction of approximately 6.59MB in parameters and a decrease of 15.0G in computational complexity. Additionally, the detection accuracy improves by 1.6%. Among these models, the proposed algorithm demonstrates the highest accuracy in detecting the appearance quality of peanut pods, with the smallest number of parameters and computational complexity, fulfilling the requirements for network lightweightness. This is conducive to the detection of peanut pods and prepares the ground for peanut quality grading.

**Table 8:** Comparison of results from different detection models.

Model	mAP0.5/%	Parameters/M	FLOPs/G	FPS/ (.s−1)	P/%	R/%	MODEL SIZE/MB
Faster R-CNN	98.7	45.98	130.4	46.3	97.2	96.4	552.9
SSD	98.4	22.63	146.7	100.2	97. 1	94.6	90.7
YOLOv7	98.3	9.33	26.7	156.2	95.4	95. 1	19.0
YOLOv8	98.8	11.13	28.4	212.7	97. 1	96.0	22.5
YOLOv5s	98.6	7.07	16.3	166.7	96.7	95.4	14.4
SSE- YOLOv5	99.3	0.48	1.3	192.3	98.3	98.5	1.1

### 3.9 Visual analysis of results

**Figure 8:**
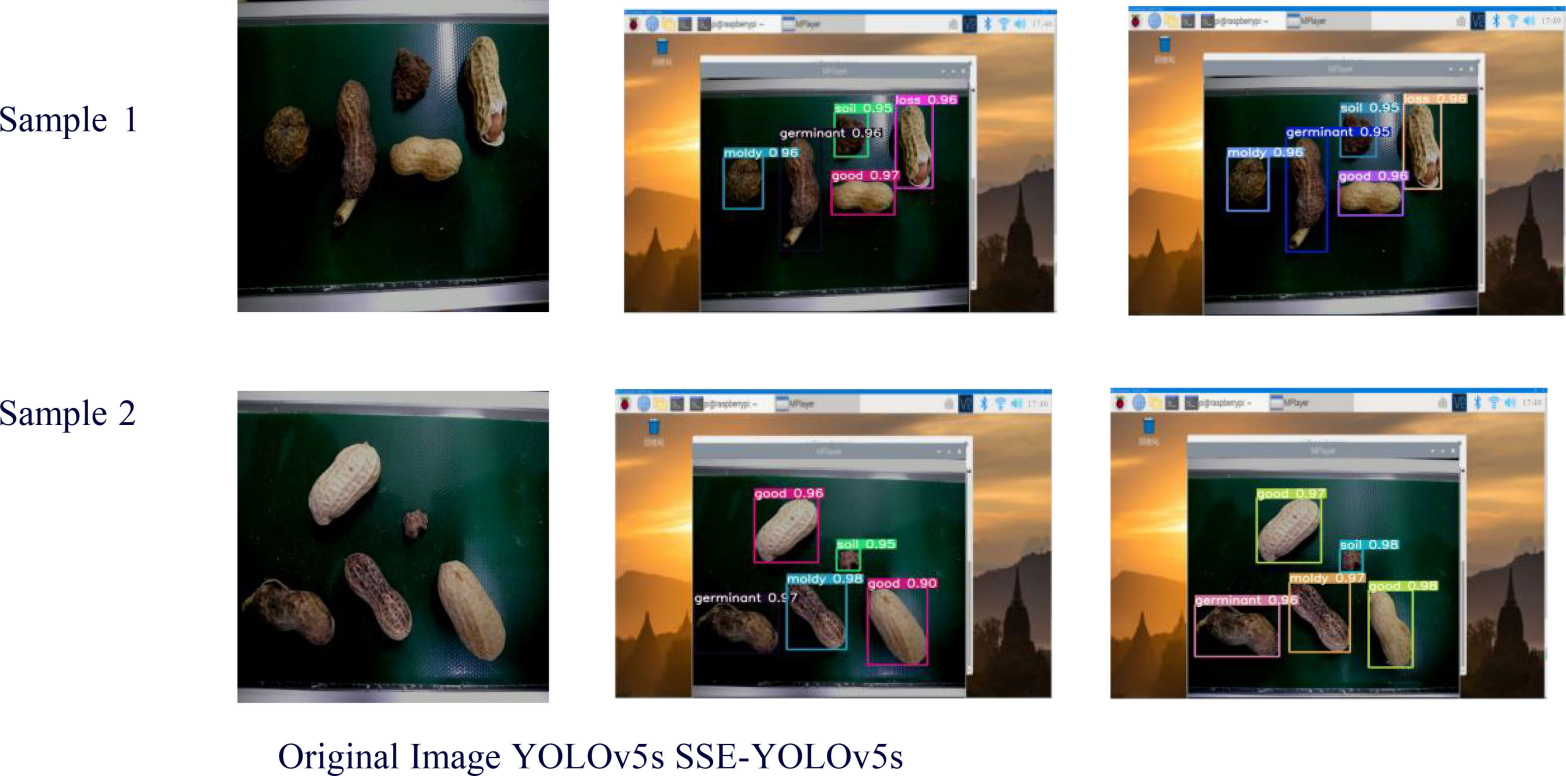
Visualization results.

To validate the efficacy of the SSE-YOLOv5s model in assessing the appearance quality of peanut pods, two sets of experimental samples were randomly selected and subjected to detection using a Raspberry Pi (a microcomputer). The visualization outcomes are presented in **Figure 8**. During the detection process with YOLOv5s, no missed detections were observed, resulting in an actual detection rate of 95.4%. Similarly, when utilizing SSE-YOLOv5s, no missed detections occurred, and the actual detection rate rose to 98.3%. During the practical testing with the Raspberry Pi, the video streams exhibited smooth playback. These findings demonstrate that the enhanced SSE-YOLOv5s model significantly accelerates the detection and recognition speed for the appearance quality of peanut pods.

## 4 Discussion

Based on the information provided, the SSE-YOLOv5s model proposed in this study offers an accurate, real-time, and secure approach for lightweight intelligent detection of five common types of peanut pod appearance quality. This model incorporates several enhancements to its components. In the backbone network, ShuffleNet v2 employs channel split and channel shuffle techniques, and is further augmented by the integration of an SE (Squeeze-and-Excitation) module. As demonstrated in **Table 4** of the experimental results, ShuffleNet v2 achieves a lightweight design (Params 0.44MB) and real-time capability (FPS 173.8). Additionally, the inclusion of the SE module, as shown in **Table 5**, further enhances detection performance (mAP 99.2%) while maintaining real-time functionality (FPS 166.9).

For the loss function, Focal-EIoU is utilized, leading to an mAP of 99. 1%, as presented in **Tables 5**. **6** showcases the superior performance of SSE-YOLOv5s compared to various detection models, particularly in terms of accuracy (mAP 99.3%), speed (FPS 192.3), and lightweight design (Params 0.48MB). **Figure 8** visually illustrates the application of SSE-YOLOv5s on a Raspberry Pi, contrasting its results with those of the YOLOv5s model, confirming the superior recognition accuracy and speed of SSE-YOLOv5s in practical applications.

Although data augmentation was adopted in this study to reduce the risk of overfitting, in practice, the model may still suffer from overfitting if the training dataset is not sufficiently diverse or small in size. To mitigate this problem, it is necessary to continuously collect more diverse sample data of peanut pods and test the model with sufficient cross-validation and generalization ability. Moreover, in real-world scenarios, peanut pods may be in complex backgrounds, such as mixed with other impurities and background colors similar to peanut pods. These complex backgrounds may cause the model to mistakenly detect the background objects as peanut pods or miss the detection of real peanut pods. To reduce the false detection rate, the model’s background suppression capability can be further optimized and more sophisticated post-processing algorithms can be considered to filter out the false detection results.

## 5 Conclusion

To address the challenges of complex detection backgrounds and high model complexity in the grading stage of peanut pods, particularly for the identification of appearance qualities such as superior, moldy, damaged, germinated, and soil-contaminated pods, this study proposes the SSE-YOLOv5s detection model, an improvement upon the YOLOv5s framework. The following conclusions are drawn:

1. The modified YOLOv5s utilizes ShuffleNet v2 as the backbone network to reduce model parameters. By embedding the SE (Squeeze-and-Excitation) attention module within the network, it enhances the focus on small targets. Furthermore, the introduction of the Focal-EIoU loss function for bounding box regression improves the model’s localization ability. The proposed algorithm achieves a detection speed of 192.3 frames per second (FPS) and a mean average precision (mAP@0.5) of 99.3%, meeting the requirements for practical applications.
2. Through experimental analysis and comparisons, the method presented in this study demonstrates significant advantages in detecting small targets compared to other object detection methods. Specifically, when compared to Faster R-CNN, SSD, YOLOv7, YOLOv8, and the original YOLOv5s algorithms, the proposed SSE-YOLOv5s model achieves the highest detection accuracy for peanut pod appearance qualities, facilitating precise detection of small targets.

## Data Availability

The original contributions presented in the study are included in the article/supplementary material. Further inquiries can be directed to the corresponding authors.
